# Novel Seed Adaptations of a Monocotyledon Seagrass in the Wavy Sea

**DOI:** 10.1371/journal.pone.0074143

**Published:** 2013-09-09

**Authors:** Keryea Soong, Shau-Ting Chiu, Ching-Nen Nathan Chen

**Affiliations:** 1 Institute of Marine Biology, National Sun Yat-sen University, Kaohsiung, Taiwan; 2 Department of Biology, National Museum of Natural Science, Taichung, Taiwan; 3 Asia-Pacific Ocean Research Center, National Sun Yat-sen University, Kaohsiung, Taiwan; University of Nottingham, United Kingdom

## Abstract

Returning to the sea, just like invasion of land, has occurred in many groups of animals and plants. For flowering plants, traits adapted to the terrestrial environments have to change or adopt a new function to allow the plants to survive and prosper in the sea where water motion tends to rotate and move seeds. In this investigation, how seeds of the seagrass *Thalassia hemprichii* (Hydrocharitaceae), a common monocotyledon in the Indo-Pacific, adapt to the wavy environment was studied. Mature seeds were collected from Dongsha Atoll in South China Sea. The effects of light qualities on seed germination, the seed morphology, the unipolar distribution of starch granules in the endosperms and growth of root hair-like filamentous cells from basal surface of the seeds were all found to differ from those of terrestrial monocotyledons. Physiologically, germination of the seeds was stimulated by blue light rather than red light. Morphologically, the bell-shaped seeds coupled with the unipolar distribution of starch granules in the enlarged bases helped maintain their upright posture on the tidal seafloor. Growth of root hair-like filamentous cells from the basal surface of the seeds prior to primary root growth served to attach onto sediments, providing leverage and attachment required by the primary roots to insert into sediments. These filamentous cells grasped coral sand but not silicate sand, demonstrating a habitat preference of this species.

## Introduction

Seagrasses are descendants of terrestrial plants that are able to complete their life cycle in the submerged photic zones in the sea. It was estimated that they entered the sea at three different times between 75 and 17 million years ago based on *rbcL* DNA sequence analysis [1], [2]. Seagrass meadows are crucial in the marine ecosystems [3], [4], [5]. They serve as nurseries and feeding grounds for diverse marine live forms. These meadows are important sites of carbon sequestration [6], and they stabilize coastline by holding sediments using rhizomes and roots. Four families of seagrasses, Hydrocharitaceae, Posidoniaceae, Cymodoceaceae and Zosteraceae are identified worldwide. These families comprise 12 genera and 58 species, and all of these species are flowering monocotyledons [1], [7]. The diversity of seagrass in different oceanic areas varies, and the Tropical Indo-Pacific bioregion has the highest diversity compared to the other five bioregions [7]. The habitat of some species such as *Thalassia hemprichii* (Hydrocharitaceae) ranges several thousand kilometers along coastline and extends to remote islands and coral reefs in open oceans [8]. On the other hand, ten species in *Phyllopadix*, *Zostera*, *Halophila*, and *Posidonia* are listed as endangered or vulnerable species in the IUCN Red List [9].

In order to propagate in the sea, ancestors of seagrasses had to adapt to new stresses imposed by the oceanic environment [10]. The first stress is the high salinity of the seawater which contains about 0.55 M salt. This level of salt is too high for the majority of terrestrial plants to stay alive. The second is the change of light spectrum. Red and far-red lights are absorbed by water easily and thus irradiation within these two wavelength ranges is scarce at the photic seafloor [11]. Red and far-red lights are environmental cues which are perceived by the photoreceptor phytochromes for many developmental programs and physiological responses in flowering plants. These developmental and physiological events include flowering, seed germination, seedling elongation, circadian rhythm and chlorophyll synthesis [12]. How seagrasses initiate these programs in an environment lacking red and far-red light is still not clear. The third significant change is the wavy environment that imposes new constraints on reproduction and dispersal processes employed by flowering plants. Seedling settlement is especially difficult in the constantly moving watery environment. In order to propagate in the sea, seagrasses must have developed mechanisms to cope with these challenges.

*Thalassia hemprichii* is a successful seagrass species in the Indo-Pacific bioregion [7], [8], [9]. However, this species is diminishing at some locations such as in the southern coast of Taiwan. For successful conservation and restoration, it is important to understand the details of how this species propagates and its habitat requirements. In this study we discovered novel developmental modifications of *Thalassia hemprichii* seeds and seedlings. These are demonstrations of *Thalassia hemprichii*’s adaptation to the wavy environment, which contribute to the success of this species in the ocean.

## Materials and Methods

### Ethics Statement

Collection permits at Dongsha Atoll National Park were granted by Marine National Park Headquarters of Taiwan. No endangered or protected species was used in this study.

### *Thalassia hemprichii* Plants, Fruits and Seeds

Plants and fruits of *T. hemprichii* were collected at Dongsha Atoll National Park of Taiwan (20°43′N, 116°42′E) between January and April in both 2011 and 2012. Seeds were gathered from the collected fruits.

### Specimen Preparation, Starch Granule Staining and Microscopy

Blocks of sectioned seeds were fixed in modified FPA fixatives (37% formaldehyde: propionic acid: 95% ethanol: glycerol: water = 1∶1∶7∶3∶8, v/v) [13]. Samples were embedded in paraffin and sectioned by a microtome. Sections of 10 µm thickness were dewaxed, rehydrated, and stained by I_2_-KI solution (1% iodine and 1% potassium iodide in water) until desired intensity was reached [14], [15]. Starch granules were observed and imaged with a light microscope using phase contrast and polarized light.

### Treatments for Seed Germination

**Figure 1:**
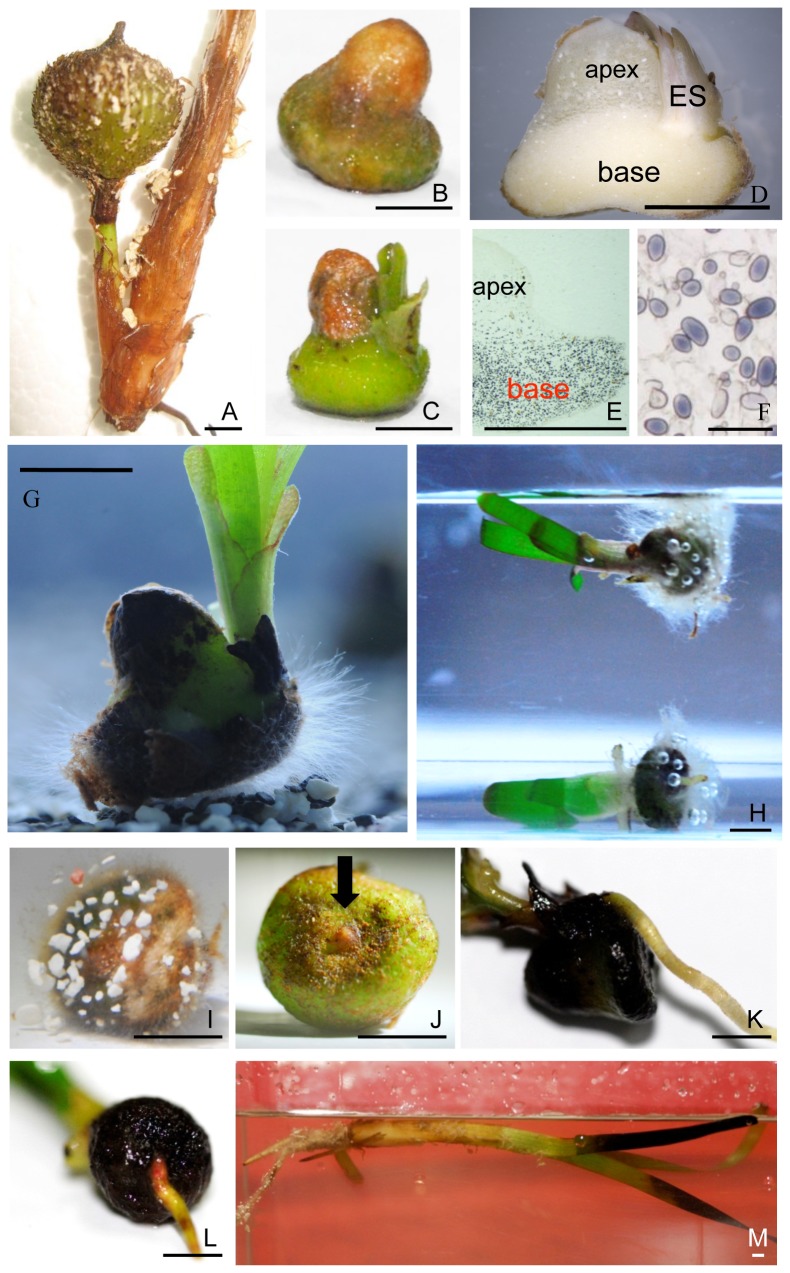
Fruit, seeds, and seedlings of *T. hemprichii*. A fruit attached to the base of a shoot (A). A typical seed with seed coat resembling a bell in shape (B). Seed germination at early stage (C). A longitudinal section of a seed illustrating texture difference between the apex and the base; ES, embryonic shoots (D). Uneven distribution of starch granules, stained by iodine in a longitudinal section, between the apex and the base (E). Magnification of the stained starch granules (F). A germinating seed with root hair-like filamentous cells emerging from all over the basal surface before emergence of primary root. Note that the sediment is composed of coral and silicate sands (G). A seedling floating in seawater when its vegetative tissue is dominant (H). A seedling right after germination shown in (G) with root hair-like filamentous cells grasping of small coral sands but not silicate sand at the seed base (I). A basal surface view of a typical seed with a tip of the primary root, indicated by the black arrow, at the base center before germination (J). A primary root emerged beneath the shoots (K). A primary root emerged from the center of seed base (L). A young plant floating in seawater (M). Scale bar in F: 100 µm; in others: 0.5 cm.

Reconstituted seawater (32 g crude salt/L H_2_O) was used for the germination tests. The tests were conducted in a darkroom at room temperature. Seed coats of these seeds were removed and they were placed in 6-well cell culture plates (well size 17×35 mm, depth and diameter, Nunc Inc.), submerged in the reconstituted seawater and illuminated continuously with intensities specified in the results. The seawater was renewed and the seeds were examined each day. Rotten and loss-of-turgor seeds were removed everyday. White light was given by an incandescent bulb. Blue and red lights were given from LEDs (light emitting diode) with spectra peaked at 455 and 645 nm, respectively. Light intensities were measured by a LI-COR photometer. Shoots reaching the height of seed tips were recorded as germinating (Fig. 1C). A total of 277 seeds were used in the germination experiments.

### Measurement of Seed and Coral Sand Densities

Total weight of a group of randomly selected nine pieces of apical or basal tissues of the seeds was measured by a digital balance. The volume of each group was measured using a 25-mL pipette (internal diameter 1.2 cm). Each group was sunk into the tip-sealed pipette filled with water. The volume of displaced water was measured by the calibrated lines on the pipette. Density was calculated as weight/volume. Density of coral sand was measured using the same principle.

## Results and Discussion

### Combination of Shape and Starch Granule Distribution in *Thalassia hemprichii* Seeds Facilitates Seed Orientation in the Wavy Sea

**Figure 2:**
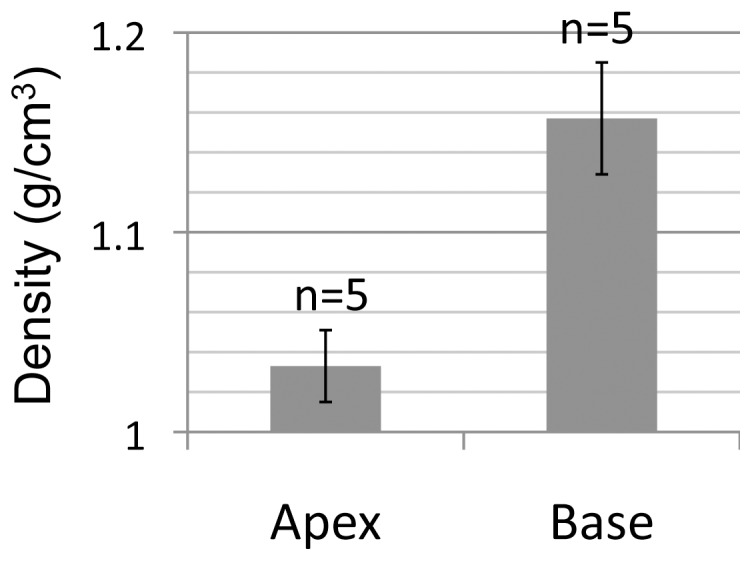
Density difference between the apex and the base of *Thalassia hemprichii* seeds. Error bars indicate 95% C.I.

The characteristic morphology of *T. hemprichii* seeds is a bell shape (Fig. 1B–D, G). In longitudinal sections, we observed that the tissue in the bases was harder and whiter than that of the apices as shown in Fig. 1D which was intriguing. By examining the microscopic structures and components of the apical and the basal cells, large amounts of starch granules were found within the base but few in the apex cells (Fig. 1E and F). The cell wall thickness of the two tissues was equivalent (data not shown). Due to the uneven distribution of starch granules in the seeds, we suspected that the tissue densities of the apex and the base would differ. To verify this assumption, the seeds were cross-sectioned at the waistline to separate the apices and the bases, and their respective densities were measured. The results showed that density of the apex tissue (1.033±0.018 g/cm^3^, mean ±95% C.I., n = 5) was at the same level as surface seawater (about 1.025 g/cm^3^). However, density of the base tissue was 12% higher (1.157±0.028 g/cm^3^, n = 5) than that of the apex tissue (Fig. 2). The bell shape of *T. hemprichii* seeds coupled with their concentrated starch granules in the enlarged bases help the seeds to adapt to the wavy environment. The function of this adaptation is to ensure at least part of the base remains in contact with seafloor and to allow the root hair-like filamentous cells growing from the basal surface to more readily grasp sediments (see below).

To the best of our knowledge, the bell-shaped seeds and the unipolar distribution of starch granules in the seeds are novel developmental modifications that have not been observed in the evolution of terrestrial plants. Anatomically, the apical and the basal tissues are parts of an endosperm that is used as nutrient reservoir for seedling growth (Fig. 1D). This is confirmed by the observations that the two tissues diminished during seedling growth and eventually disappeared. Each monocotyledon seed contains one embryo and one endosperm, which result from double fertilization. Endosperm cells are triploid, and their genetic activities are permanently switched off when seeds reach maturity. Since the apical and the basal cells are both parts of an endosperm, they are generated from the same triploid progenitor cell. How the differentiation of the two tissues is regulated during the seed developmental process needs to be clarified through further studies.

### Growth of Root Hair-like Filamentous Cells from the Basal Surface of the Seeds Prior to Primary Root Emergence Prevents Rolling of the Seeds Caused by Moving Seawater

An additional novel developmental modification of the seeds for the adaptation to the wavy environment is that root hair-like filamentous cells grow before emergence of the primary root during seed germination. As shown in Figure 1G–I, the filamentous cells grew from all over the basal surface of the seeds at an early stage of germination. These filamentous cells were able to grasp coral sand but not silicate sand, presumably by attaching to the small grooves and holes of coral sand grains or due to chemical interactions (Fig. 1I). The density of coral sand was 2.544±0.016 g/cm^3^ (n = 5) by our measurements, which was much higher than that of the seeds. The growth of the filamentous cells prior to primary roots prevents the seeds from rolling on seafloor in the wavy environment. These cells also provide the gripping power necessary to allow primary roots to insert into compact coral sand beds.

Gravity is detected by a mechanism comprising specialized starch granules called amyloplasts in the root tip cells in flowering plants [16]. In a seed being rolled by currents, the amyloplasts are agitated constantly which prevents the root tips from identifying the gravity vector and thus gravitropic growth is abolished. This would likely lead to death of the plant if its primary root is not able to grow in the right direction to anchor the new seedling.

For terrestrial plants, root hairs are extensions of root epidermal cells, which grow at the maturation zone of a root. The identity of the cells that generate the root hair-like filamentous cells from the basal surface of *T. hemprichii* seeds has yet to be positively identified. However, evidence suggests they are aleurone cells. Aleurone is a layer of tissue covering the surface of endosperm. These cells and endosperm cells share the same origin. However, endosperm cells are filled with starch and have little biochemical activities. In contrast, aleurone cells contain high level of protein and are biochemically active during seed germination [17]. In grass seeds such as corn, rice, barley and wheat, aleurone cells secrete enzymes into endosperm during seed germination to digest the starchy tissue. The degraded biomaterials are then used as nutrients for seedling growth. Since the endosperm cells are degraded by aleurone cells during germination, it is unlikely this cells could develop structures such as these root hair-like filamentous cells. This suggests that the cells growing the filamentous cells are likely aleurone cells. More studies are required to verify this hypothesis. These filamentous cells perished eventually together with the aleurone and endosperm cells as the seedlings continued their growth.

### *Thalassia hemprichii* Seed Germination was Enhanced by White and Blue Light but not Red Light

As mentioned previously, red and far-red wavelengths are scarce at the seafloor. It is uncertain whether the red/far-red light photoreceptor phytochromes in *T. hemprichii* have lost their function since no selection pressure was imposed on the photoreceptors over the course of its evolution. Seed germination is a typical test used to examine the function of phytochromes. In most plant species, red light is able to stimulate seed germination even at low levels. On the other hand, mutants with impaired phytochrome function are not able to perceive red light, resulting in significant reduced germination rates compared to wildtype counter parts [18].

**Figure 3:**
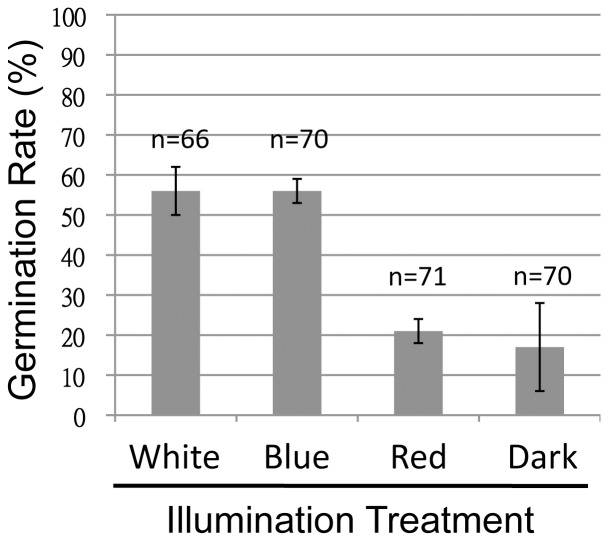
White and blue light enhances germination of *Thalassia hemprichii* seeds. Seeds were immersed in the reconstituted seawater and illuminated under different light treatments. The light intensities were set at 50 µmol/m^2^s. “n” indicates combined number of seeds in duplicated tests. The experiments were terminated when the germination rate of at least one group exceeded 50%. Error bars indicate 95% C.I.

*T. hemprichii* seeds were germinated under various light conditions. While over 50% of seeds illuminated under white or blue light at 50 µmol/m^2^s germinated, only 21% of seeds treated under red light of the same intensity germinated. This low percentage was comparable to seeds germinated in darkness which was 17% (Fig. 3). The differences between white/blue and red/dark were all significant (P<0.01, *t*-tests). To exclude the possibility that the low germination rates under red light was caused by the low light intensity, the germination was re-examined under 50 and 200 µmol/m^2^s of red light. The results showed that there was no difference between the two treatments over the course of germination (data not shown). Based on these results, it is very likely that the phytochrome or its downstream signaling genes in *T. hemprichii* have mutated, resulting in a loss of either red light perception or the signaling pathways. Cloning and sequencing of these genes will be necessary to verify these hypotheses.

*T. hemprichii* seeds are non-dormant, *i.e.,* their germination process starts within a short period of time after seed maturation. Nevertheless, they are still able to fine tune the timing of germination based on the availability of white and blue light wavelengths that are more abundant than red light on the seafloor. However, blue light has rarely been related to seed germination in terrestrial plants. Blue light, as an environmental signal, is perceived by the photoreceptors cryptochromes and phototropins. The canonical physiological roles of blue light receptors include regulation of circadian rhythm, phototropism, and stimulation of stomatal opening [19]. How blue light stimulates *T. hemprichii* seed germination is currently unclear.

### *Thalassia hemprichii* Seedlings Anchored to Coral Sand but not Silicate Sand

**Figure 4:**
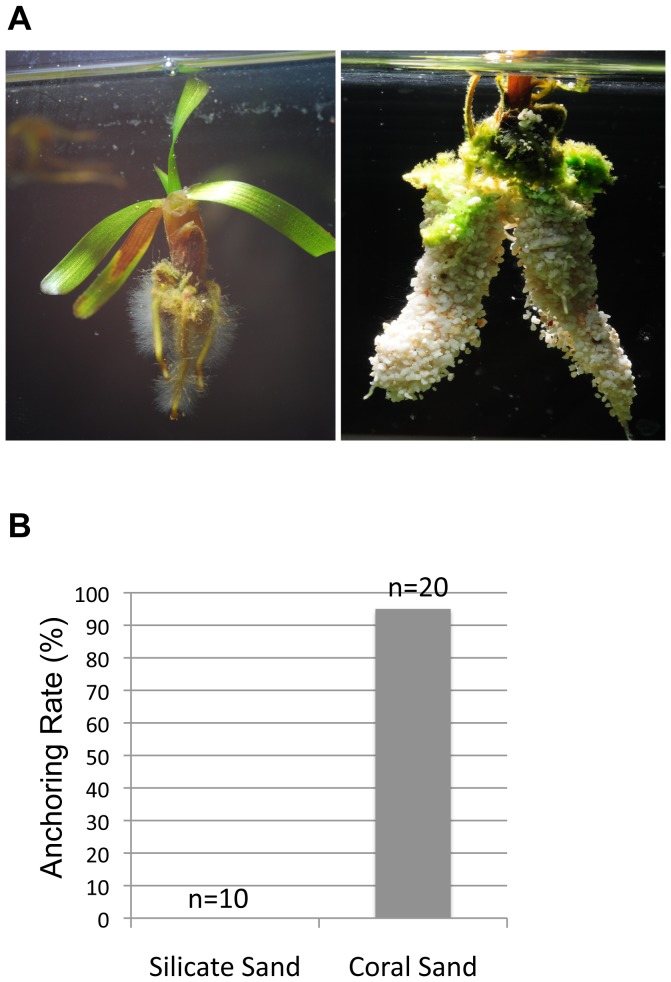
*Thalassia hemprichii* seedlings anchor to a bed of coral sand but not silicate sand. Seeds were allowed to germinate on beds of silicate or coral sand in the same aquarium. Seedlings anchored themselves using root and root hairs to grip coral sand (A, right and B) but not silicate sand (A, left and B; *Chi*-square test, p<0.01). Seedlings not anchored were floating in the water column when the vegetative tissues became dominant (A, left).

We observed that most *T. hemprichii* habitats are close to coral reefs or on dead reef platforms rather than on silicate sand beds. In addition, *T. hemprichii* population is declining in the southern coast of a national park in Taiwan which has become a stretch of silicate sand beach. The two lines of evidence suggest the possibility that the seeds have difficulty in anchoring to a silicate sand bed. To test whether the root hair-like filamentous cells could distinguish sediments, *T. hemprichii* seeds were germinated on (1) a bed with mixed coral and silicate sand as shown in Figure 1G, and (2) silicate or coral sand bed separately in the same aquarium that were divided by a porous plate and illuminated under 100 µmol/m^2^s white light 16 hours a day. In the first experiment, the filamentous cells from the basal surface grew first as described previously and they held onto coral sand only as shown in Figure 1I but not silicate sand. In the second experiment, the roots of the coral sand group were able to insert into the bed successfully. On the other hand, the roots in the silicate sand group were unable to insert into the sediment. The seedlings in this latter group eventually floated to the top of the water column when nutrients in the endosperms were depleted and its vegetative tissues became dominant (Fig. 4A, left). Plenty of root hairs were growing from the roots (Fig. 4A, left), which is a typical trait of flowering plants. A seedling of the coral sand group was pulled out of the sediment to demonstrate the holding capacity of the root hairs to coral sand (Fig. 4A, right). The anchoring rates of the seeds to the sediments are dependent on the composition of the sand, as 19 out of 20 seeds in coral sand secured to the sediment but all 10 in the silicate sand group floated (Fig. 4B).

We have discovered three novel developmental modifications of the monocotyledon seagrass *Thalassia hemprichii*, which had not been observed among terrestrial plants. These modifications include the bell-shaped seeds, the unipolar distribution of starch granules in the base of the seeds and the root hair-like filamentous cells growing from the basal surface of the seeds prior to primary roots. The roles of these modifications are to increase the probability of the seedlings to anchor into coral sand beds in the wavy environments. In addition, we also discovered that red light is no longer able to stimulate seed germination of *T. hemprichii* but white and blue light do. How this species executes the signal of blue light to stimulate seed germination is an interesting question. *T. hemprichii* seedlings have a preference for coral sand beds but not silicate sand. This helps to explain the difficulty of restoring *T. hemprichii* population in the black sandy coastal areas.

Unanchored *T. hemprichii* seedlings and plants are able to float in seawater (Figs. 1H, 1M, and 4A left). This may provide an alternative way of dispersal for this species other than using seeds, if the transported seedlings and plants are able to anchor themselves onto sediments at their destinations. Whether the plants have similar preference over sediment composition for anchoring as germinating seeds needs to be clarified.
